# Neural network based corrosion modeling of Stainless Steel 316L elbow using electric field mapping data

**DOI:** 10.1038/s41598-023-40083-y

**Published:** 2023-08-11

**Authors:** Azhar M. Memon, Imil Hamda Imran, Luai M. Alhems

**Affiliations:** Applied Research Center for Metrology, Standards, and Testing, Research Institute, King Fahd University of Petroleum and Minerals, Dhahran, 31261 Saudi Arabia

**Keywords:** Mechanical engineering, Techniques and instrumentation, Electrical and electronic engineering

## Abstract

Stainless steel (SS) is widely employed in industrial applications that demand superior corrosion resistance. Modeling its corrosion behavior in common structural and various operational scenarios is beneficial to provide wall-thickness (WT) information, thus leading to a predictive asset integrity regime. In this spirit, an approach to model the corrosion behavior of SS 316L using artificial neural networks (ANNs) is developed, whereby saline water at different concentrations is flown through an elbow structure at different flow rates and salt concentrations. Voltage, current, and temperature data are recorded hourly using electric field mapping (EFM) pins installed on the elbow surface, which serve as training data for the ANNs. The performance of corrosion modeling is verified by comparing the predicted WT with actual measurements obtained from experimental tests. The results show the exceptional performance of the proposed single ANN model to predict WT. The error is calculated by comparing the estimated WT and actual measurement recorded, where the maximum error for each setting is range from 0.5363 to $0.7535\%$. RMSE and MAE values of each pin in every setting are also computed such that the maximum values of RMSE and MAE are 0.0271 and 0.0266, respectively. Moreover, a concise account of the observed scale formation is also reported. This comprehensive study contributes to a better understanding of SS 316L corrosion and offers valuable insights for developing efficient strategies to prevent corrosion in industrial environments. By accurately predicting WT loss using ANNs, this approach enables proactive maintenance planning, minimizing the risk of structural failures and ensuring the extended sustainability of industrial assets.

## Introduction

Pipelines and other infrastructure form the backbone of the global economy. It is of utmost importance to ensure their structural integrity in order to prevent downtime and disruptions in the supply chain. They play a crucial role in transporting fluids and are vital infrastructure for various sectors, including hydroelectric, marine, nuclear power plants, food processing, and oil and gas industries. However, these pipelines are susceptible to issues like corrosion, dents, defects, and cracks, which can lead to failures and pose significant safety risks. Such failures can result in leaks, ruptures, fatal accidents, environmental damage, and financial consequences such as expensive repairs, outages, and production delays. To mitigate these risks, it is essential to prioritize the maintenance and integrity of pipeline assets by implementing regular inspections and maintenance practices. As a result, the field of pipeline corrosion inspection, evaluation, modeling, and prediction has gained significant attention in both academic and industrial settings. This focused area of study aims to develop effective methods and tools to assess and predict pipeline corrosion, enabling proactive measures to prevent failures and ensure the continued safe operation of these critical infrastructure systems^1–5^.

Corrosion is the most frequent phenomenon and serious pipeline failure mechanism^3^, which significantly shortens the operational life of pipelines. It may come in various forms, including general or uniform^6^, pitting, crevice, inter-granular, erosion–corrosion (E–C), corrosion brought on by bacterial activity, and environment-induced cracking. The corrosion rate in the pipeline is associated with external and internal factors. Some examples of external factors are workplace environment, soil composition and condensation for buried pipelines, or water chemistry for underground pipes. Meanwhile, several internal factors causing corrosion are flowing liquid activity, type of fluid transported, temperature, flow rate, and tension of the fluids^7^. In this context, accurately measuring the loss of wall thickness in pipelines in real-time and during operation becomes a critical task. This is particularly relevant for SS pipes, which are prone to flow accelerated corrosion and scaling caused by harsh fluids.

This work focuses on the modeling and prediction of E–C in SS elbows, taking into account the presence of a saline solution. Stainless steel, despite being an expensive and heavy alloy, offers numerous advantages that outweigh its drawbacks in various applications and industries. SS is widely preferred in many fields due to its extensive range of types and grades, its versatility in different applications, and its ability to resist UV rays and corrosion. It is known for its durability, strength, and food-grade status, making it suitable for high-temperature environments. Industries such as food and beverage rely on SS due to its resistance to microorganism growth, while wastewater treatment equipment benefits from its ease of maintenance. Furthermore, an interesting advantage of SS hardware is that it retains its monetary value even if there are changes in operating procedures or the plant is salvaged. This adds to its appeal in different settings. In the following section, we will provide a brief overview of notable works that have been conducted on the subject of E–C in SS.

Certain coatings can be used to protect SS surfaces from corrosion and prevent or reduce the degradation of pipes. These coatings act as a protective layer and are applied to the SS surface. However, over time, these protective layers can be stripped away from the SS surface due to friction from sand or other corrosive agents present in the fluid. This process is known as E–C and can accelerate corrosion. If E–C occurs in conjunction with fluid flow dynamics, it can lead to significant thinning of the pipe walls. In fact, E–C can cause wall infiltration at a greater rate compared to corrosion or erosion alone. Piping elbows, in particular, are highly susceptible to this phenomenon and are considered the most vulnerable part of the piping system^8^.

In this sense, semi-conductivities of passive films on the surface of SS 304 elbow were investigated in saline fluid containing sand over a loop system^9^. The mixture included 0.9 wt$\%$ sand particles (size of 400–500 $\upmu$m) and 3.5 wt$\%$ NaCl. The semi-conductivities and materials of passive films using spectroscopy were reported in regard to the impacts of flowing fluid at the elbow. E–C of a carbon steel (CS) elbow in a CO2 environment with sand particles as part of the flow loop was reported in^10^. There were three characteristics relying on the fluid velocity discovered such as the formation of protective scales at low velocities yielding low rates of corrosion, the prevention of scales formation at higher velocities generating higher and more uniform rates of corrosion, and localized points were monitored along with the cumulative formation of protective scales with deep pits at intermediate velocities. Furthermore, a computerized model for predicting sand erosion in pipes was provided to estimate the sand erosion in the pipelines. The environmental levels over which E–C is useful to estimate pipeline material thickness loss were investigated^11^. In particular, the degradation of pipeline steel (API X65) owing to E–C with flowing fluid containing sand in a CO2-saturated atmosphere was studied. E–C prediction was improved by adding environmental or external parameters, such as temperature, fluid flow rate, and solid loading, to the empirical, amechanistic, and computational models.

Some results to evaluate the degradation rate in 90$^{\circ }$ elbows were studied in various scenarios. Numerical simulations were carried out for a 90$^{\circ }$ elbow with air–sand–water flow under a pseudo-slug flow erosion setting^12^. The finding indicated that the top of the elbow suffers the most erosion compared to the other parts for the horizontal direction. Some experimental tests were conducted to study erosion in a 90$^{\circ }$ elbow with numerous fluid flows^13^. The results showed that tees and elbows are the most prone parts to experience erosion. A high material loss caused by E–C was studied in^14^ for 3D-printed 90$^{\circ }$ elbow carbon steel. A study comparing the E–C characteristic of CS 1018 and SS 304L for 90$^{\circ }$ long-radius elbows in slug flow settings containing sand particles was reported in^15^. The findings revealed that SS had a smoother surface than CS, CS elbow E-C rate was four times greater than SS elbow E–C rate, and the E–C rate on the top half of the elbow was higher than the lower half for both materials. As a result, it can be concluded that SS has excellent corrosion resistance in comparison to CS.

The SS loss was predicted for nuclear-grade SS 316 in high-temperature sodium conditions using sodium corrosion rate equation^16^. This equation was constructed by some elements such as the impact of temperature, sodium oxygen levels, and sodium velocity. Corrosion estimation was studied for SS 316L under flow-accelerated corrosion (FAC)^17^. A mass transfer investigation was carried out by introducing a corrosion model into the numerical study. The prediction values for pipe sections with abruptly variable diameters were compared to the measured ones. Nevertheless, the predicted corrosion depth was approximately 1.3 to 3.5 times the average experimental corrosion depth for the uniform part.

Several approaches have been proposed to predict and detect defects and failures in the field of pipeline integrity assessment. An overview of the use of machine learning techniques for evaluating the integrity of corroded oil and gas pipelines was presented in^18^. Various pipeline corrosion aspects such as corrosion detection, prediction of remaining useful life, and risk assessment were discussed. Some applications of machine learning in assessing the integrity of corroded oil and gas pipelines were explored. A corrosion model was developed in^19^ to predict pipeline defects due to the failure pressure in the pipeline using ANN. The training data were generated from the finite element method (FEM). Full-scale burst tests were used to validate before conducting various pipeline failure simulations with numerous corrosion geometry features and loadings. The prediction errors achieved by ANN were between $-9.39$ and $4.63\%$ with a coefficient of determination ($R^2$) of 0.9921 when compared with the finite element analysis (FEA) results. The same approach was also proposed in^20^ with a different presentation.

The phenomenon of stray current corrosion in Q235A steel and the developing prediction model for this type of corrosion was studied in^21^. Through experimental analysis, the corrosion behavior of Q235A steel was examined under varying chloride concentrations and stray current densities. The aim was to investigate the corrosion mechanisms and identify key factors influencing the process. Another approach was proposed in^22^ to identify a crack in the pipeline by integrating a deep learning algorithm and 3D shadow modeling (3D-SM). The crack shadow below the crack area utilizing light sources was projected. This approach was verified in the experimental test and has $93.53\%$ accuracy and a $92.04\%$ regression rate to identify the crack.

For corrosion data modeling and knowledge mining, a deep structure model called densely connected cascade forest-weighted K nearest neighbors (DCCF-WKNNs) was investigated in^23^. The effectiveness of the model was evaluated using a dataset comprising 409 outdoor atmospheric corrosion samples of low-alloy steels. By combining random forests-K nearest neighbors RF-WKNNs and DCCF-WKNNs, the proposed approach surpassed commonly employed machine learning algorithms like ANN, support vector regression (SVR), RF, and cascade forests (cForest) in terms of accurately predicting corrosion rates in this study. Additionally, the model showed the capability to forecast corrosion rates under varying environmental conditions by considering individual factors such as pH, temperature, relative humidity, SO$_2$, rainfall, or Cl$^-$. In summary, the determination coefficients ($R_2$) of the proposed methods are between 0.785 and 0.924 with ANN and DCGF-WKNNs as the worse and best results, respectively.

The ANN-based model was proposed in^24^ by considering various parameters into account such as pipe material properties, defect characteristics, and operating conditions to predict the failure pressure of buried high-strength pipes. The model was trained using a dataset generated from a numerical simulation of the pipe’s response to internal pressure and stray current corrosion. The results demonstrated that the proposed ANN model can effectively predict the failure pressure of the pipes with a high level of accuracy. The application of SVM and ANN methods for assessing the vulnerability of urban buried gas pipeline networks was studied in^25^. The study found that both SVM and ANN methods effectively assessed the vulnerability of the gas pipeline network, with SVM demonstrating slightly higher accuracy. The findings of this research contribute to improving the understanding of vulnerability assessment methods for gas pipeline networks and can be useful for decision-making in urban planning and risk management. ANNs models to predict the kinetics of gas hydrate formation in multiphase systems was proposed in^26^. The experiments were conducted using a pure system and a multiphase system with crude oil to analyze the rate of formation in both cases. The results showed that the addition of crude oil influenced the rate and mole consumption of gas hydrate formation, with the multiphase system forming hydrates faster due to the non-Newtonian behavior of crude oil at high pressure and low temperature. The prediction models demonstrated satisfactory performance, with high R2 values close to 1 and low MSE values close to 0.

The remaining life of the pipeline was predicted using historical operational data in^27^. The recurrent neural network (RNN) model was trained on various input parameters such as operating pressure, temperature, and corrosion rate. The study demonstrated the effectiveness of the proposed approach by comparing its predictions with actual data from a dry gas pipeline. The RNN model performs well during training, testing, and validation stages, with high R2 values and low mean square error (MSE) values. The model accurately predicts the pipe’s condition and corrosion defects even when certain input parameters are missing. Additionally, the RNN model effectively estimates the pipe life based on different parameters, such as wall thickness and pressure. ANN model was developed in^28^ using real-time risk inspection data to predict the remaining useful life of piping based on parameters such as pressure, corrosion, wall thinning, age, thickness, outer radius, and product type. The model demonstrated strong performance with an $R^2$ value of 0.99 and a validation accuracy of $97.51\%$. Corrosion was found to have the highest impact on the piping system, while age had the lowest impact.

The study focused in^29^ on comparing different machine learning algorithms, including ANN, support vector machines (SVM), and decision trees (DT), to determine their effectiveness in predicting corrosion risk. Some experiment evaluations were conducted using a dataset comprising corrosion-related parameters and comparing the performance of the machine learning models. The results showed that the ANN model outperforms the SVM and DT models in terms of accuracy, precision, and recall. The ANN model demonstrated its capability to effectively predict corrosion risk and provides valuable insights for corrosion management strategies. An approach for fault diagnosis in natural gas compressors was proposed in^30^ by using vibration analysis, adaptive stochastic resonance (ASR), and generative adversarial networks (GANs). The objective was to overcome the challenges of detecting faults in complex and nonlinear vibration signals by introducing controlled noise through ASR and enhancing fault features using GANs. The combined ASR-GAN approach is validated using experimental data and demonstrates improved accuracy and efficiency compared to traditional methods. SR performance was evaluated by measuring its accuracy range between 93.42 and $97.26\%$ under various parameter settings. Another development was presented in^31^ for predicting the life condition of crude oil pipelines. ANN was proposed to create the predictive model. The study focused on addressing the challenges associated with monitoring and maintaining the integrity of crude oil pipelines, which are critical for oil transportation. Various factors affecting pipeline degradation, such as corrosion rate, pressure, temperature, and time were considered for modeling. The pre-processed data and train the ANN model were conducted utilizing a backpropagation algorithm. The results demonstrated that the ANN-based model has the capability to predict the life condition of crude oil pipelines based on the input parameters.

Due to the large span of pipeline systems, it is critical to determine and predict the remaining WT of SS caused by a corrosive environment. Even though there are several investigations conducted on corrosion in the SS pipeline. However, the extensive ANN model for the complex corrosion and passive layer formation inside an SS 316L elbow taking into account water salinity and flow dynamics is deficient in the literature. Surface corrosion of 316L steel was elaborated using ANN and statistical analysis in 5 wt% NaCl solution^32^. Some 2D grayscale images were presented to illustrate the corrosion morphology.

From the aforementioned literature, it becomes possible to predict the extent of wall thickness loss in advance by utilizing NNs. This forecast capability greatly assists in maintaining pipeline facilities. Furthermore, by training similar models for specific applications, it can assess the associated risks. The methodology finds a wide range of applications, including the food industry, nuclear reactors, water desalination, and more. Recently, an initial result of the ANN model for WT of SS 316L elbow as the section of a running saline water loop was reported by the authors^33^. However, it was developed for wall thinning prediction with a single flow rate and salt concentration. By extending the results in^33^, a more general ANN model SS 316L elbow flown by saline water with various flow rates and concentrations is studied in this paper. The target is to have a single ANN model representing the corrosion behavior. It is constructed using voltage, current, and temperature measurements recorded hourly by the experimental setup consisting of EFM pins attached to the elbow section. According to the best authors’ knowledge, this is the first investigation used to develop an ANN model for SS elbow with various flow rates and salt concentrations. Moreover, this paper concisely describes a comprehensive chemical and microscopic analysis of scales formed on the internal surfaces.

The remainder of the paper is organized as follows. The experimental setup is presented in next section. Following that, the materials and methods of the experiments are explained in next section. The modeling part includes brief literature surveys of NN, training set, and corrosion modeling presented in next section. Then in “Results”, the ANN model is tested under various settings illustrated using several figures and a table to show the quantitative performance. The conclusion and direction for future work are presented in the last section.

## Experimental setup

**Figure 1:**
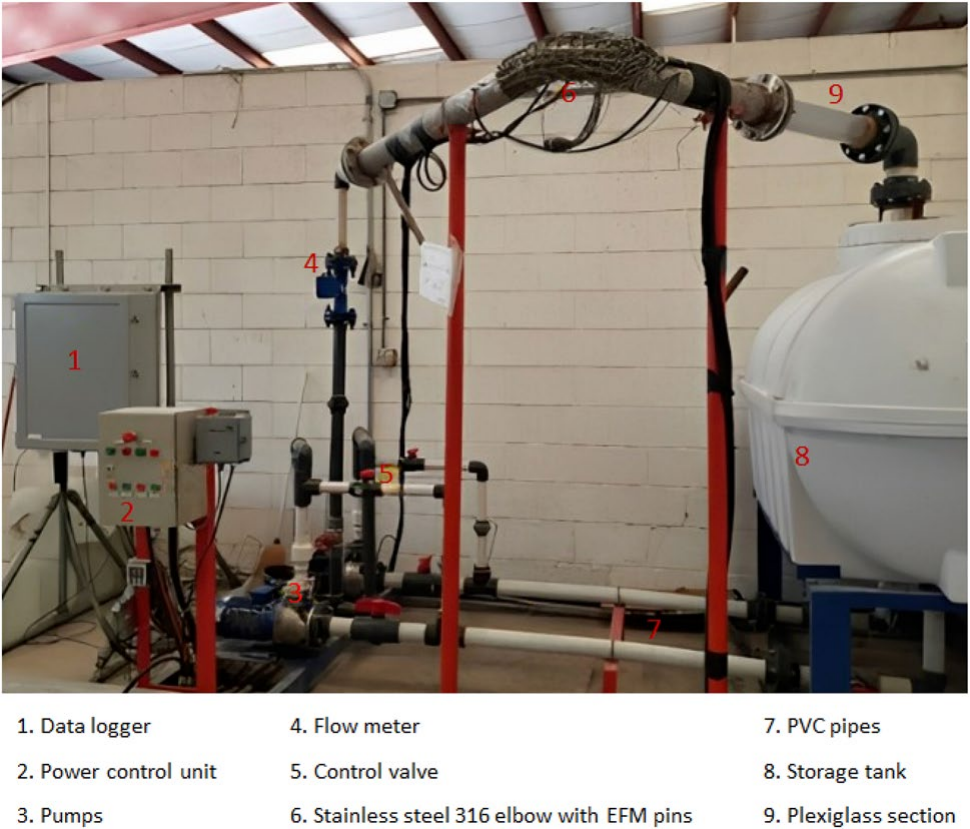
Experimental setup.

**Figure 2:**
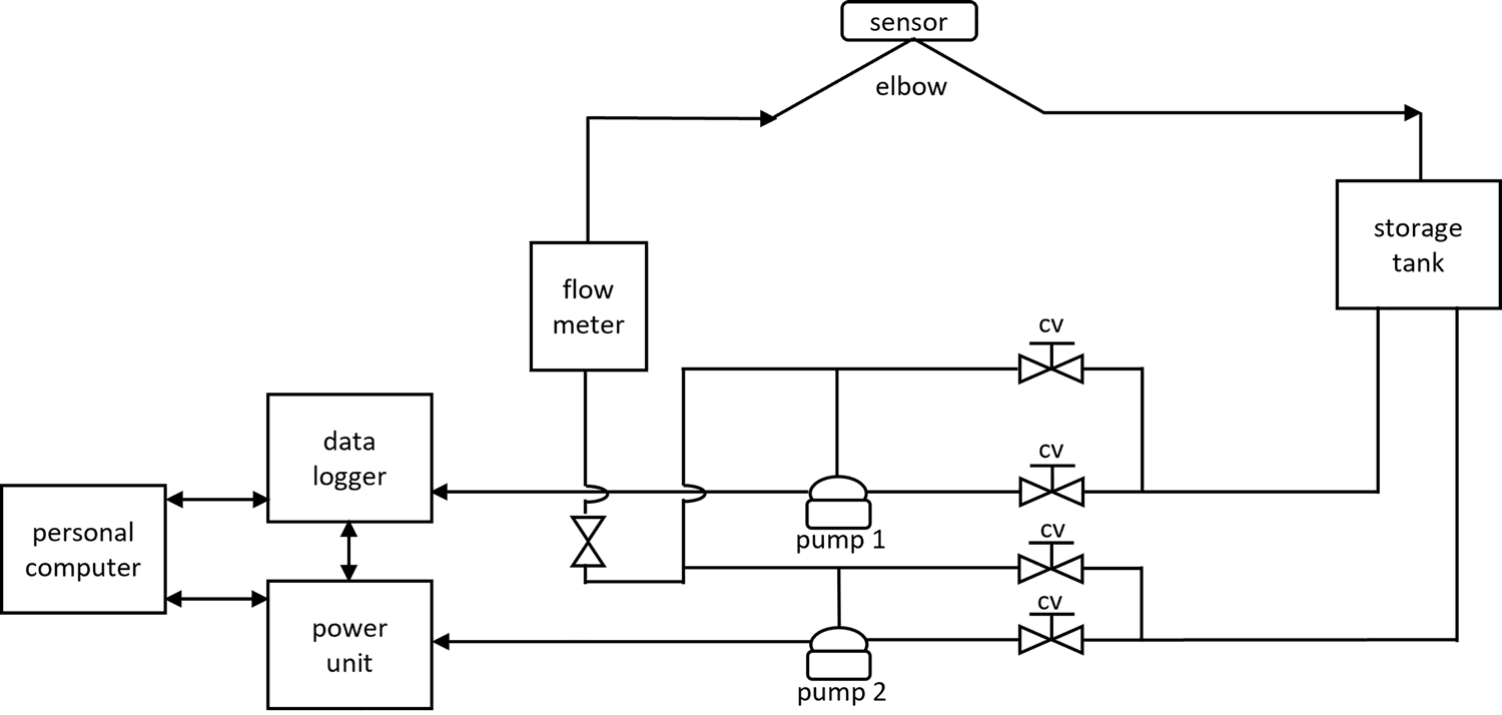
Schematic diagram of mini flow loop.

**Figure 3:**
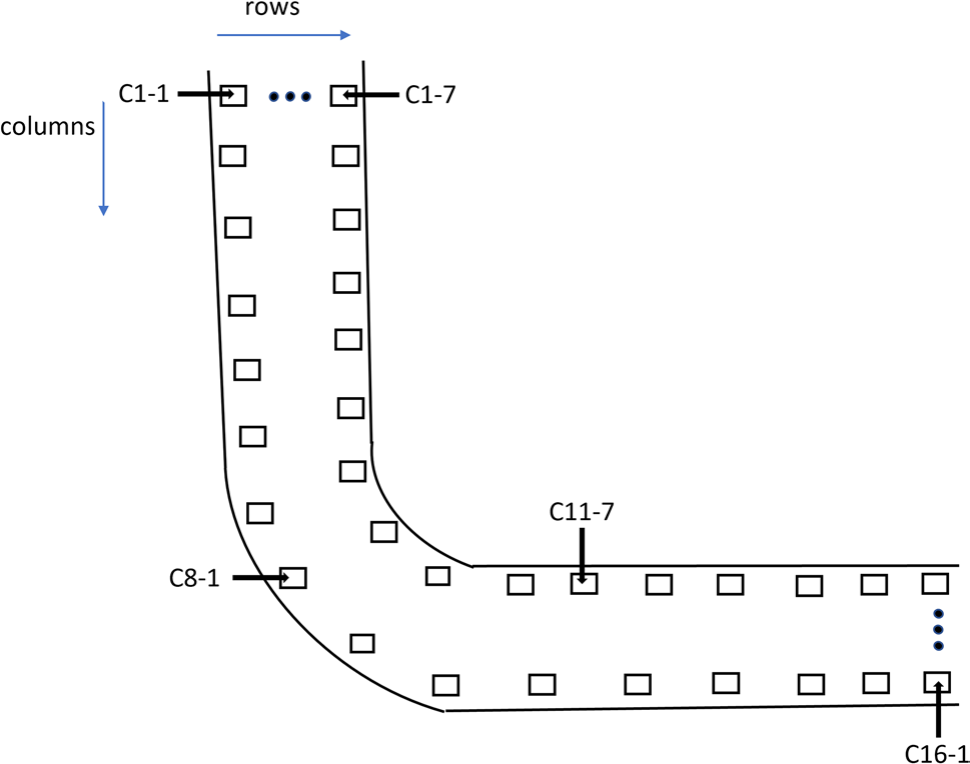
Illustration of the elbow pipe showing positions of 16 columns by 7 rows pin positions.

In this section, the experimental setup consisting of a state-of-the-art flow loop representing the real-time industrial conditions is presented as illustrated in Fig. 1. It has two centrifugal pumps from Lowara Company, model number TG334, flow rate (Q) 45 m$^3$/h, head (h) 110 m, OMEGA turbine flow meter (FTB730), power switch (Eurotherm 2500P Schneider Electric), Plexiglass pipe section for inspecting the flow condition visually, and elbow section of SS 316L. This section is 3.01 m long with 4 in. internal diameter and a nominal wall thickness WT of 6.03 mm. The flow inlet and outlet arms of the elbow are 1.2 and 1.25 m long (including 0.6 m long Plexiglass), respectively. The inlet arm, elbow section, and outlet arm of the pipe elbow are all made of corrosion-resistant SS 316L. The schematic and column layout of the flow loop can be seen in Figs. 2 and 3.

EFM monitor with an array of non-intrusive sensing pins are permanently attached to the surface of the elbow in the matrix of 16 columns and 7 rows, i.e., a total of 112 pins, as illustrated in Fig. 3. These pins have non-intrusive characteristics and do not penetrate the pipe. From the pins, the measured amount of excitation current and simultaneous measurements of the voltage pattern injected into the structure is recorded. The collected data are composed of a time series of differential pin voltages (in $\upmu$V) and the corresponding array of current shunt measurements (in Amperes). The resistance of a volume is oppositely related to the WT, where a thinner wall contains a higher resistance to current flow. As a result, it generates a higher voltage drop for the same amount of excitation current. An extra temperature compensation step is added to account for the change in resistivity due to the existing temperature variations resulting from the fluid flow and ambient conditions. Supplemental pins for temperature measurements are utilized for this purpose. The system is configured to read electrical data and compute the remaining WT of the elbow section. Nevertheless, the manufacturer does not provide this calculation software. The workflow scheme is that data is uploaded to their server, and then they provide all calculations and the WT results after several weeks. This situation inspires us to develop a local NNs-based model to estimate the WT using the available voltage, current, and temperature measurements in relatively less time.

## Materials and methods

Pure dried vacuum (PDV) salt was thoroughly dissolved in 1 m$^3$ of tap water without sand to evaluate the effect of simulated seawater on corrosion and scale deposit. The salinity of the prepared solution with various concentrations and flow rates is measured using a handheld refractometer (REF234), as shown in Table 1. The fluid was drained from a 2 m$^3$ storage tank and flown using both centrifugal pumps, as presented in Fig. 1. These pumps have a maximum combined flow rate of 70 m$^3$/h. Control valves were deployed to manage the flow rates at the pump’s entrance and exit. An inline turbine flow meter was used to measure the rates and total flow pumped.

**Table 1:** Flow rate and salinity concentration data.

	Flow rate (m/s)	Concentration	No of samples for each pin
Scenario 1	2	3.5	358
Scenario 2	1	3.5	361
Scenario 3	1	3	121
Scenario 4	2	4	352

At the start, the experimental data included baseline measurements of the pipe WT taken with an ultrasonic testing (UT) probe as well as hourly recorded differential voltage ($\Delta V$), differential current ($\Delta I$), and temperature (*T*). The manufacturer provided a server to feed the pipe thickness reading prior to the experiment. ANN is deployed to train $\Delta V$, $\Delta I$, and *T* measurements as the training input and the WT of the pipe WT delivered from the manufacturer’s analysis software as output data. UT probing reported elevated WT as a result of scale formation and a decrease in WT after scale removal from the pipe at the end of the experiment, as explained in Section “Scale formation” and “WT monitoring”.

### Scale formation

**Figure 4:**
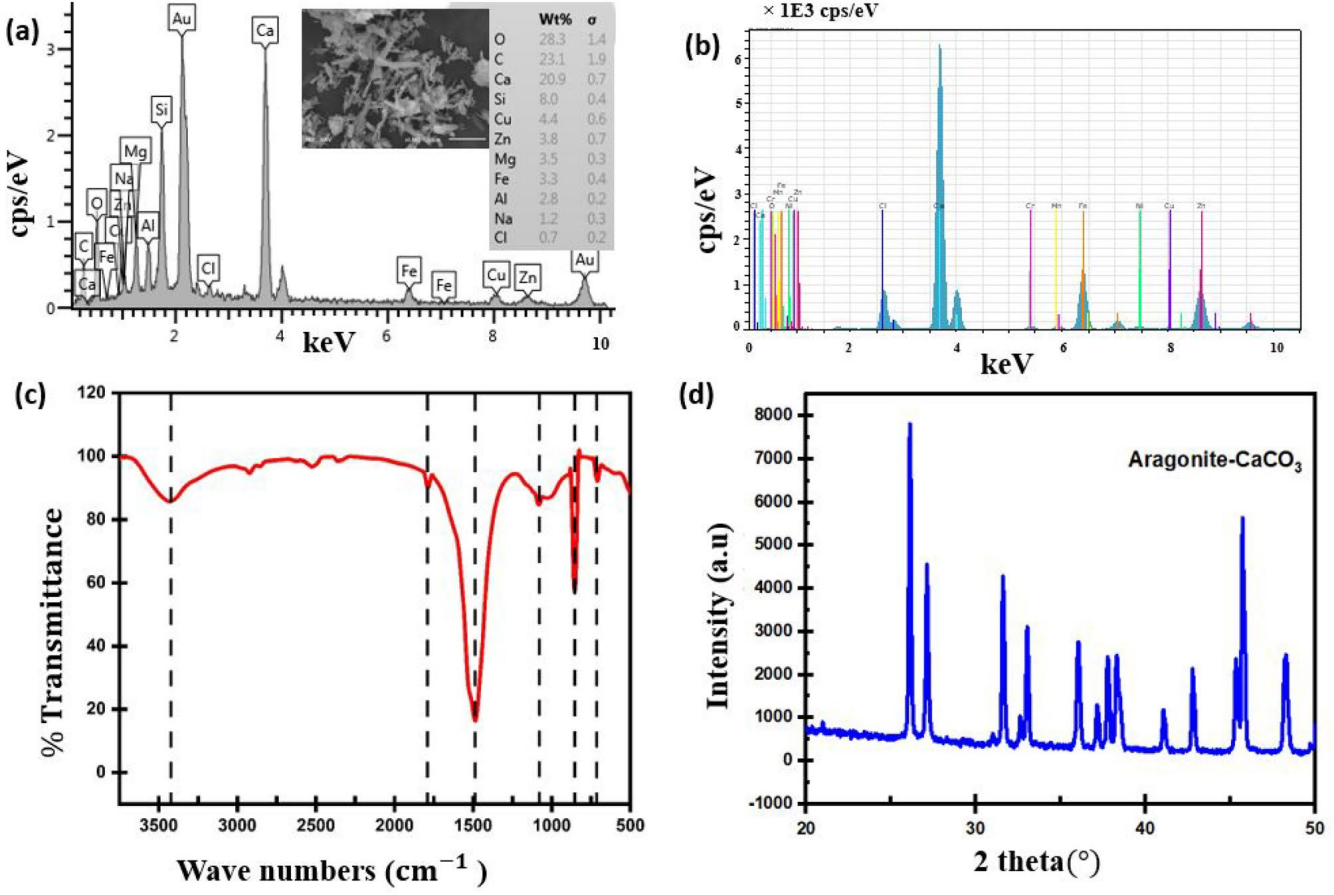
Spectroscopic analysis of extracted scale deposit; (**a**) SEM-EDS; (**b**) XRF; (**c**) FT-IR; (**d**) XRD. The figure was reproduced with permission from our previous work^33^.

Spectroscopic analysis was conducted on the scale deposit sample retrieved from the pipe during cleaning. The composition of the water running through the system was discovered to be the cause of scale formation, as it contained calcium with a concentration of 291 mg/L. Figure 4a depicts the percentage elemental composition of the extracted sample obtained from scanning electron microscopy with energy dispersive spectroscopy (SEM-EDS) characterization. The X-ray fluorescence shown in Fig. 4b represents the oxide state of the elements not captured by EDS analysis. The Fourier-transform infrared spectroscopy (FT-IR) spectrum of the aragonite-CaCO3 scale illustrated in Fig. 4c has distinctive peaks at 709.1, 854.3, and 1486.7 cm$^{-1}$ due to the C–O stretching and bending modes. As shown in Fig. 4d, the sharp peak of the X-ray diffractogram is typical of a crystalline compound with $2\theta$ values ranging from $25^{\circ }$ to $50^{\circ }$. The short peak at $2\theta$ of $35^{\circ }$ was unexpected and suspected to be foreign materials of contaminants in the sample. The presence of a high crystalline nature indicates that the CaCO3 scale is in the aragonite phase. This was verified by comparing the SEM micrograph of the sample shown in Fig. 4a with the study reported in^34^.

### WT monitoring

**Figure 5:**
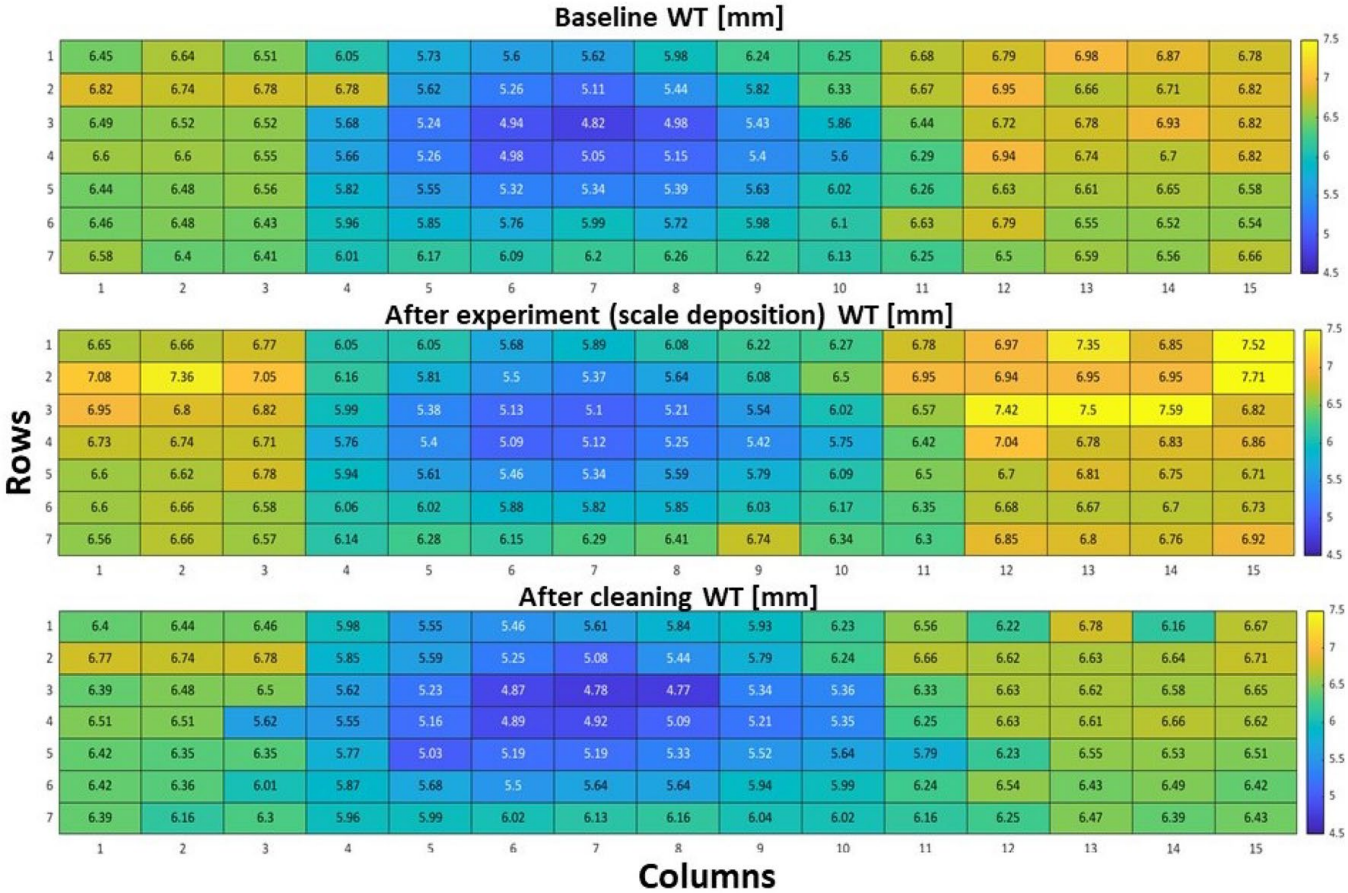
Pipe WT color map before the experiment (top), after the experiment (middle), and after cleaning (bottom). The figure was reproduced with permission from our previous work^33^.

**Figure 6:**
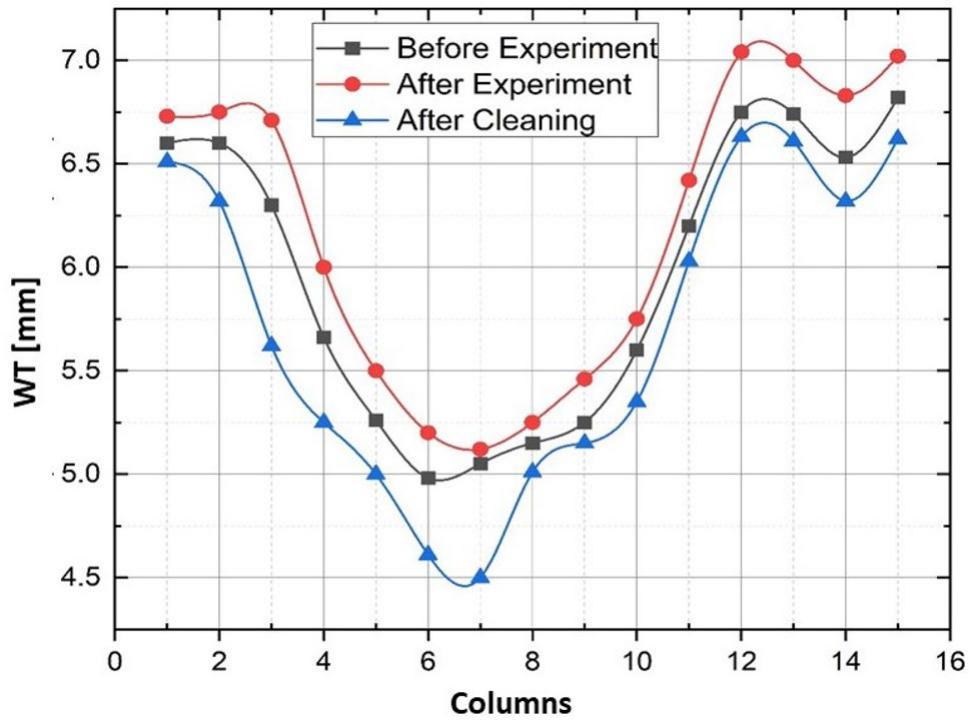
Pipe WT color map before the experiment (top), after the experiment (middle), and after cleaning (bottom). The figure was reproduced with permission from our previous work^33^.

These readings were taken externally with the UT gauge to monitor the change in WT as the experiments progressed. The same matrix as EFM pins was utilized to confirm that subsequent measurements were performed on the same points. The results for baseline or reference WT, after the experiment to assess the deposition of scale, and after cleaning for wall loss measurement are illustrated in Fig. 5. The data collected revealed an average $3\%$ increment in WT and a maximum raise of approximately $13\%$ due to scaling. WT data obtained after a thorough cleaning with the strong-pressure washer was also evaluated by comparing it to the baseline, showing a mean of $2.6\%$ and a maximum $14\%$ reduction. These measurements and observations are in conformance with the findings published in the literature^35–37^. It stated that wall thinning is severe at the bend compared with other parts of the pipe because of sudden changes occurring in the flow direction and velocities. Figure 6 shows the WT distribution at the center of the matrix outlay (Row 4). It is illustrated in the graph that the elbow section thickness increases after six months of exposure to the solution due to the scale deposit on the WT of the elbow. After cleaning, the WT decreased below the baseline, with the greatest thickness loss recorded near the middle of the elbow section (Row 4, point 7) of the pipe.

## Modeling

This section begins with a background of previous works related to corrosion modeling using NNs, their types and a comparison is given. Following that, the description of the data sets and NN used are presented.

### Background

Modeling in particular applications with complex processes, such as corrosion, is extremely difficult, if not impossible, and beyond the scope of conventional machine learning algorithms. In this situation, NNs composed of neurons structured as input, output, and single or multiple hidden layers come into play. These layers serve as the basis for learning hidden patterns in the existing data. The number of hidden layers and neurons grows in proportion to the complexity of the data. Interested readers are directed to^38^ for a thorough discussion on the fundamentals, operation, and structure of NNs, which has been left out of this section for breviary. A probabilistic NN, generalized regression neural network (GRNN), was employed in^39^ to model and predict the corrosion potential and current densities of CS samples in soils with various parameters that significantly impact the corrosion rate of steel. Moreover, the soil parameters were subjected to sensitivity analysis. ANNs were utilized to predict the pitting corrosion of SS 316L and EN 1.4404, while environmental conditions were taken into account in^40,41^. The results in the marine environment, in particular, were later reported.

The pitting corrosion in stainless steel was explored utilizing statistical and ANN modeling of AISI 316 LVM passivation process, which aims to provide a passive protective layer on the steel surface for biomedical applications^42^. In this case, pitting corrosion and passivation were used as variables for the models. It was determined that only ANN provided precise predictions with a low mean relative error compared to statistical approaches. Some interesting results to predict the pitting corrosion for AISI 316 using ANN, for example in^43^ corrosion modeling was developed by considering various environmental variables and in^44^ with different classification models. The pitting corrosion of SS 304 by seawater containing Cl for construction purposes was discussed in another work^45^. By considering the variables that influence this process, stochastic models were designed to estimate the life of the corroding steel structure last for. The parameter that defines the onset of a leakage, i.e maximum pit depth, was particularly modeled. It was observed that it is primarily determined by the electro-chemical driving force and the time elapsed.

A comprehensive study on the modeling for predicting stress corrosion cracking (SCC), specifically in structural materials used in nuclear power plants, was recently published in^46^. Numerous references were presented and discussed, with positive outcomes of using ANNs for prediction. Numerical simulations were conducted in^47^ to explore the SCC propagation behavior for a specific scenario of a stainless steel 316L elbow with an extremely high WT (about 80 mm) used in nuclear reactors. An artificial defect was proposed on the internal surface to imitate the actual crack.

### Types and comparison of NNs

There are several types of NNs that can be employed depending on the application requirements^38^. The most widely utilized are perceptron, feed-forward (FF), convolution, recurrent, Kohonen maps, and support vector machines (SVM). The most fundamental and smallest NN is perceptron, which performs specific computations to detect some features in input data. This type can only be implemented for the solutions of linearly separable problems due to its simple structure.

Meanwhile, FF NNs can be used in more complicated applications such as speech processing, image processing, and other computer vision applications. This method can be divided into single-layered and multi-layered NNs. In this case, the number of layers varies according to the system’s complexity. Additionally, it has the capability to deal with significant amounts of noisy data and is quick and simple to implement. Convolutional NNs, on the other hand, are difficult to build and perform slowly depending on the number of hidden layers.

Recurrent NNs is an approach that can be proposed to model sequential data. It can be applied for more advanced tasks such as grammar checking, translation, text auto-suggest, and text-to-speech. Nevertheless, training data can be complicated. Kohonen maps can be employed in specific applications to recognize patterns in data, for example, in medical analysis to categorize data. SVMs have to be highly reliable in various prediction applications including regression, classification, and outliers detection.

### Description of the training set

**Figure 7:**
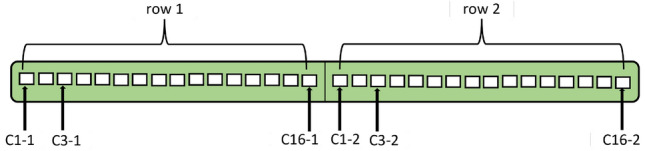
Illustration of data restructuring for NN training.

The training inputs used in this work are $\Delta V$, $\Delta I$ collected from an array with 7 rows and 16 columns following the EFM pin pattern. The temperature (*T*) is measured at the 10 spots on the SS elbow. It means that there are 234 measurements that can be used for training data. The measurements are recorded hourly with various flow rates and concentrations for several days providing 121–358 measurements for every parameter, as shown in Table 1. Each row was opened separately and concatenated with the next one to create the training input model for NNs, resulting from 7 rows and 16 columns in a total of $(16~\text {columns} \times 7~\text {rows} = ) 112$ series. Figure 7 depicts the structure of the first two rows as an example of data restructuring, where the hours were converted into rows.

The flow rate 2 m/s saline water with concentration $4\%$ measurements (scenario 4) is used as training data. There are 352 measurements for each pin recorded hourly. Therefore, the dimension of each training input was from 352 by 234 for $\Delta V$, $\Delta I$, and *T*, resulting in a 352 by 234 matrix for all the parameters.

The targets were determined by measuring the remaining WT values between the pin locations, therefore resulting in $(15~\text {spots between the pin columns} \times 7 \text {rows} = ) 105$ samples of remaining WT monitored for 482 h. It means that the dimension of the target was 482 by 105. This data is distributed for NN modeling, $70\%$ of data was allocated for training, $15\%$ for validation, and $15\%$ for testing. In other words, 246, 53, and 53 measurements were selected for training, validation, and testing, respectively. To avoid constant observations of WT measurements that can terminate the training process, white noise with zero mean and standard deviation $5\times 10^{-3}$ *mm* is applied to the WT data during the training. This value is less than 0.1% of the nominal WT.

### NN for corrosion modeling

Due to the aforementioned benefits, FF NN was chosen for this study from the NNs discussed above. Furthermore, it was intended to begin with the most basic NN after Perceptron, i.e., the FF network, and progress to more advanced ones if the corrosion process was not adequately modeled. The model was trained using supervised learning due to the availability of input and output training data sets. In this research, the Levenberg–Marquardt algorithm is used for modeling SS corrosion. The Levenberg–Marquardt algorithm is an optimization algorithm commonly used for training NNs. It is an extension of the Gauss–Newton algorithm, which is used for solving nonlinear least squares problems. The Levenberg–Marquardt algorithm combines the advantages of the Gauss–Newton method and the gradient descent method to efficiently find the optimal weights of a neural network. Unlike conventional methods that require computation of the exact Hessian matrix, this algorithm operates using the gradient vector and the Jacobian matrix^48–51^. A loss function defined as a sum of squared errors is expressed by

1 $$\begin{aligned} f = \sum _{i=1} ^{m} e_i^2, \end{aligned}$$

where *m* is the number of training samples.

The Jacobian matrix of the loss function can be defined as a matrix that consists of the derivatives of the errors with respect to the parameters as represented by

2 $$\begin{aligned} J_{i,j} = \frac{\partial e_i}{\partial w_j}, \end{aligned}$$

for $i=1 \ldots n$ and $j=1 \ldots m$, where *m* is the number of samples in the data set and *n* is the number of parameters in the neural network.

The gradient vector of the loss function is generated by

3 $$\begin{aligned} J_{i,j} = \nabla f = 2 J^{T} e, \end{aligned}$$

where $$e=\begin{bmatrix} e_1&\cdots&e_m \end{bmatrix}^{T}$$ represents all error terms. The following expression is used to approximate the Hessian matrix

4 $$\begin{aligned} H f \approx \nabla f = \lambda I + 2 J^{T} J, \end{aligned}$$

where $\lambda$ is a damping factor to ensure its positivity, and *I* is an identity matrix. The Levenberg–Marquardt algorithm defines the process of improving the parameters as expressed by

5 $$\begin{aligned} w_{i+1} = w^i - (\lambda _i I + 2 J_i^{T} J_i)^{-1} ( 2 {J_i}^{T} e^{i} ), \end{aligned}$$

where $i=0, 1 \ldots$. From the above information, it can be seen that when the damping parameter $\lambda$ is set to zero, the method being utilized is essentially Newton’s method, employing an approximation of the Hessian matrix. Conversely, if $\lambda$ is assigned a large value, the method transitions into gradient descent with a low learning rate.

The initial value of the parameter $\lambda$ is set to a large value, ensuring that the initial updates in the gradient descent direction are small. If an iteration fails, $\lambda$ is increased by a certain factor. On the other hand, as the loss function decreases, $\lambda$ is decreased, allowing the Levenberg–Marquardt algorithm to gradually approach the Newton method. This iterative process generally enhances the convergence speed towards the minimum value. The first step of the training process of ANN using the Levenberg–Marquardt algorithm is by computing the loss, the gradient, and approximating the Hessian matrix. Following that, the damping parameter is tuned to minimize the loss in every iteration.

The architecture of the Marquardt–Levenberg algorithm can be summarized as follows

- Initialization: The algorithm starts by initializing the parameter vector to some initial values. These initial values can significantly impact the convergence and accuracy of the algorithm. Careful initialization based on prior knowledge or heuristics is often required.
- Jacobian matrix: The algorithm calculates the Jacobian matrix, which represents the partial derivatives of the objective function with respect to the parameters. The Jacobian provides information about the sensitivity of the objective function to changes in the parameters.
- Gauss–Newton step: The algorithm performs a Gauss–Newton step by approximating the objective function as a quadratic function around the current parameter values. It solves a linear system of equations to estimate the update for the parameters. However, this step can be sensitive to ill-conditioned problems, where the Hessian matrix is poorly conditioned or not invertible.
- Levenberg–Marquardt step: To address the sensitivity issue in the Gauss–Newton step, the algorithm introduces the Levenberg–Marquardt step. It modifies the linear system of equations by adding a damping term to the diagonal elements. This damping term controls the trade-off between the Gauss–Newton and gradient descent steps, as discussed earlier.
- Parameter update: The algorithm updates the parameter vector based on the computed step size obtained from either the Gauss–Newton or the Levenberg–Marquardt step.
- Convergence check: After each parameter update, the algorithm checks if the convergence criteria are met. If the criteria are satisfied, the algorithm terminates; otherwise, it proceeds to the next iteration.

To evaluate the performance of ANN, the coefficient of determination ($R^2$), root mean square error (RMSE), and mean absolute error (MAE) are computed using the following equations

6 $$\begin{aligned} R^2&= 1- \frac{\sum _{i=1} ^{n} (y_i-\hat{y}_i)^2}{ \sum _{i=1} ^{n} (y_i-\bar{y})^2 } \end{aligned}$$

7 $$\begin{aligned} \text {RMSE}&= \sqrt{\frac{1}{n} \sum _{i=1} ^{n} (\hat{y}_i-y_i)^2} \end{aligned}$$

8 $$\begin{aligned} \text {MAE}&= \frac{1}{n} \sum _{i=1} ^{n} | \hat{y}_i-y_i | , \end{aligned}$$

where $y_i$ and $\hat{y}_i$ are the actual and estimated *i*-th outputs with *n* sampling data, respectively. The average of actual outputs is denoted by $\bar{y}$.

The input is denoted to be $x_i$, $i=1,2,\ldots ,n$ where $n=234$, such that each of length 352 results in the training input size of 234 by 452. The input parameters were divided into three categories, where EI $(x_1,x_2,\ldots ,x_{112})$, EV $(x_{113},x_{114},\ldots ,x_{224})$, and T $(x_{225},x_{226},\ldots ,x_{234})$. On another side, the output was denoted by $y_i=y_1,y_2,\ldots ,y_{105}$.

The MATLAB neural network toolbox has means to adjust the network variables or parameters to improve learning accuracy^52^. There are some parameters that can be adjusted to increase the learning performance such as the number of epochs that can be selected by default provided by NN toolbox. While others such as the number of neurons and size of hidden layers can be chosen by trial and error experience^53^. The error and average error to evaluate the effectiveness of the model are computed using (9) and (10), respectively.

9 $$\begin{aligned} \text {error}&= \frac{|\text {estimated WT}-\text {measured WT}|}{\text {measured WT}} \times 100\% \end{aligned}$$

10 $$\begin{aligned} \text {average error}&= \frac{\sum _{i=1}^N |\text {error}|}{N}, \end{aligned}$$

where *N* is the number of data points.

There are 352 measurements recorded hourly in scenario 4 as presented in Table 1. The size of layers is selected to be 10 using random data division and the Lavenberg–Marquardt training algorithm. The validation performance achieved is $2.6520\times 10^{-5}$ at $11{\text {th}}$ epoch and R values of 1.00, 1.00, and 1.00 are achieved for training, validation, and test sets, respectively. This implied that the selected model had outstanding learning capabilities. The detail of the training model can be seen in Table 2.

**Table 2:** NN training to map predictors using Lavenberg–Marquardt algorithm.

	Observations	MSE	R value
Training	246	$2.3648\times 10^{-5}$	1.00
Validation	53	$2.6520\times 10^{-5}$	1.00
Test	53	$2.7001\times 10^{-5}$	1.00

## Results

**Figure 8:**
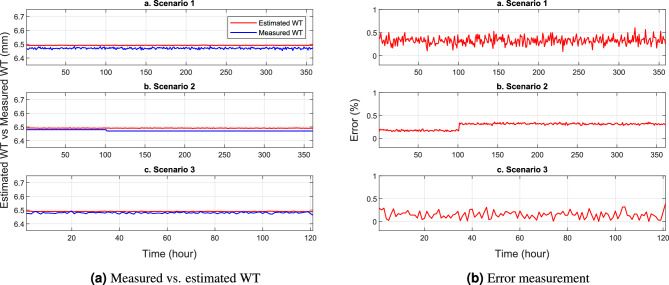
WT prediction at pin C1-3.

**Figure 9:**
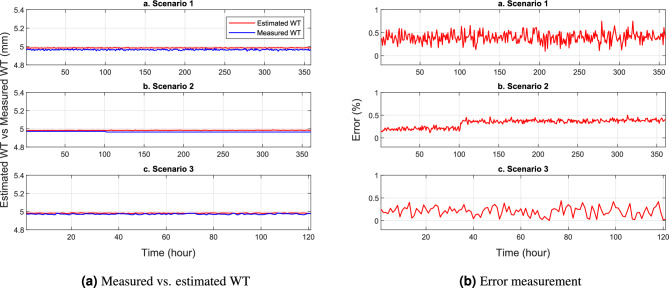
WT prediction at pin C8-3.

**Figure 10:**
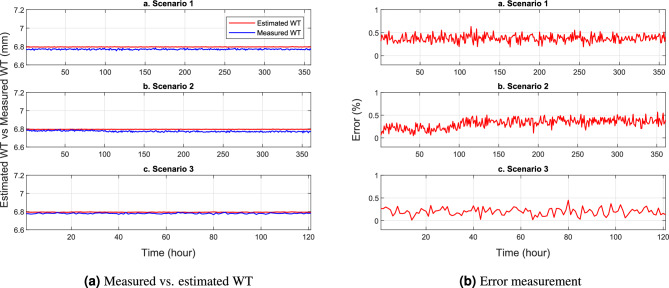
WT prediction at pin C13-6.

This section presents several numerical tests showing the performance of the trained NNs model in predicting the remaining WT. In Figs. 8, 9 and 10, a comparison is made between the estimated WT values and the measured ones, focusing on scenarios 1, 2, and 3 at pin locations C1-1, C8-3, and C13-6, respectively. The results highlight an interesting observation that the WT of the SS elbow varies within the same row but in different columns. Specifically, it is evident that the remaining WT around the ends of the elbow arm (pin C1-3 and C8-3) is thicker than the center of the elbow (pin C8-3). Figures 8a, 9a and 10a provide a visual representation of this phenomenon. The difference in WT distribution is attributed to the manufacturing process of the pipe. Nevertheless, despite these variations, the NN model shows its robustness y providing highly accurate predictions with minimal errors in estimating the remaining WT.

The provided visualizations offer valuable insights into the relationship between the predicted and actual WT values. By examining the different scenarios and pin locations, it becomes evident that the remaining WT is not uniform across the SS elbow. The observed disparity in thickness, especially between the ends and the center of the elbow, can be attributed to the manufacturing techniques employed during the production of the pipe. Despite this non-uniformity, the trained NN model demonstrates its effectiveness by accurately estimating the remaining WT. The small errors in prediction indicate the model’s capability to handle these variations and provide reliable estimations.

**Figure 11:**
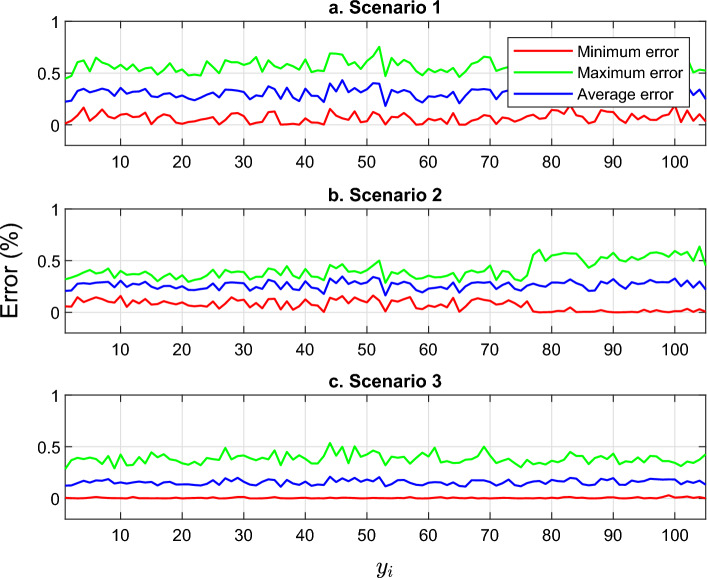
The minimum, maximum, and average errors for three scenarios given in Table 3.

Figure 11 provides a comprehensive overview of the minimum, maximum, and average errors associated with each pin in the WT prediction. This visualization allows for easy comparison and analysis of the NN model’s performance across different pins. It can be seen that the NN model demonstrates its ability to accurately predict the WT values under varying flow rates and concentrations. To facilitate a more convenience presentation of the errors, Table 3 presents the errors for each pin across scenarios 1, 2, and 3 involving all 112 data points. The table clearly illustrates the exceptional accuracy of the NN model in estimating WT measurements across a range of flow rates and concentrations. Remarkably, the maximum error observed is extremely small i.e. $0.7535\%$ in scenario 1.

These findings emphasize the robustness and reliability of the NN model when it comes to predicting WT values in diverse conditions. By considering multiple scenarios and incorporating a wide range of data points, the model demonstrates its ability to consistently generate accurate estimates. Even under challenging circumstances involving different flow rates and concentrations, the NN model maintains a high level of accuracy. This is evident in the minimal and maximum error values presented in Table 3, further validating the effectiveness and precision of the NN model in WT prediction tasks.

**Table 3:** Error between measured and estimated WT.

	Maximum error ($\%$)	Minimum error ($\%)$	Average error ($\%$)
Scenario 1	0.7535	$3.6981\times 10^{-4}$	0.3077
Scenario 2	0.6359	$1.0500\times 10^{-4}$	0.2639
Scenario 3	0.5363	$1.0624\times 10^{-6}$	0.1570

RMSE and MAE are also calculated for scenarios 1, 2, and 3 for further evaluation, as presented in Table 4. This table presents the maximum, minimum, and average values of RMSE and MAE computed from each EFM pin. The results from these calculations highlight the exceptional performance of the NNs in estimating the remaining WT of SS elbows. All the values demonstrate the outstanding ability of NNs in this regard.

**Table 4:** RMSE and MAE of ANN corrosion modeling.

	RMSE			MAE		
	Maximum	Minimum	Average	Maximum	Minimum	Average
Scenario 1	0.0271	0.0105	0.0197	0.0266	0.0093	0.019
Scenario 2	0.0233	0.0091	0.0171	0.0222	0.0084	0.0163
Scenario 3	0.0146	0.0073	0.011	0.0135	0.006	0.0097

The findings presented above show that NN modeling is a valuable tool for the in-depth analysis of pipe corrosion characteristics. These results highlight the potential of utilizing NN models to gain comprehensive insights into the behavior of pipes. In particular, the study emphasizes the importance of understanding the elbow section of a pipe that can be used for the construction of NN models. By incorporating all the necessary measurements obtained through the principle of EFM, a trained NN model can be developed. This approach holds promise for accurately modeling and predicting corrosion in pipelines. From this study, it can be concluded that the NN model could contribute significantly to the field of pipe corrosion research and facilitate proactive maintenance and management strategies.

## Conclusion

This paper studied the initial findings of an artificial neural network (ANN) model applied to predict corrosion in an SS 316L elbow exposed to saline water with different flow rates and concentrations. The measurement setup included a SS 316L elbow with EFM pins attached to measure the voltage and current readings, as well as temperature. These measurements were carried out for a period of 352 h and were used to train and test the ANN model. The trained model accurately estimated wall thickness (WT) using readings obtained at various flow rates and concentrations. The maximum errors computed over the entire elbow section were $0.7535\%$, $0.6359\%$, and $0.5363\%$ under scenarios 1, 2, and 3, respectively. Moreover, the RMSE and MAE of all pins in every scenario were calculated with the maximum value of RMSE being 0.0271 and the maximum value of MAE being 0.0266. The promising results obtained motivate further exploration and implementation of an online ANN-based tool for real-time prediction of remaining WT in SS elbows. Also, it will be interesting to compare other machine learning approaches to estimate the remaining WT of various SS elbow in future works. This tool would offer valuable insights for maintenance and integrity management in industrial applications.

## Data Availability

The datasets generated and/or analyzed during the current study are not publicly available due to the confidentiality but are available from the corresponding author on reasonable request.
